# Roctavian withdrawal: exploring the gap between innovation and healthcare system readiness in hemophilia A gene therapy

**DOI:** 10.1016/j.rpth.2026.106881

**Published:** 2026-07-28

**Authors:** Matteo Nicola Dario Di Minno, Flora Peyvandi

**Affiliations:** 1 Department of Clinical Medicine and Surgery, University of Naples Federico II, Naples, Italy; 2 Department of Pathophysiology and Transplantation, Università degli Studi di Milano, Milan, Italy; 3 Hemostasis & Thrombosis Unit, Angelo Bianchi Bonomi Hemophilia and Thrombosis Center, Fondazione Istituto di Ricovero e Cura a Carattere Scientifico Ca’ Granda Ospedale Maggiore Policlinico, Milan, Italy

**Keywords:** cost-benefit analysis, genetic therapy, health care economics, health services accessibility, hemophilia A

## Abstract

The voluntary withdrawal of valoctocogene roxaparvovec (Roctavian) represents an inflection point in hemophilia A gene therapy. Notably, this decision was not driven by safety or efficacy concerns, but by challenges to economic sustainability, market access, and healthcare system readiness. This paper examines the clinical evidence supporting gene therapy, including durability and safety, and highlights how uncertainty in long-term transgene expression complicates both clinical management and economic evaluation. The Roctavian case is not isolated but reflects a broader pattern observed across gene therapies, where high costs, limited uptake, and system-level constraints have led to market withdrawal or restricted access. These dynamics reveal a gap between scientific innovation and healthcare delivery, suggesting that the barriers to gene therapy are not biological but structural. Addressing this gap will require not only technological advances but also sustainable reimbursement models, coordinated long-term follow-up infrastructure, and healthcare systems capable of supporting advanced therapies beyond their approval.

## 1 Introduction

The withdrawal of valoctocogene roxaparvovec (Roctavian, BioMarin Pharmaceutical Inc) is a critical inflection point in hemophilia A gene therapy, not due to safety or efficacy limitations but to systemic constraints in access, sustainability, and healthcare system readiness [1]. This distinction underscores a growing disconnect between scientific progress and healthcare system preparedness (Figure). Bridging the gap between the promise of advanced therapies and the real-world readiness of healthcare systems remains a major challenge.

**Figure:**
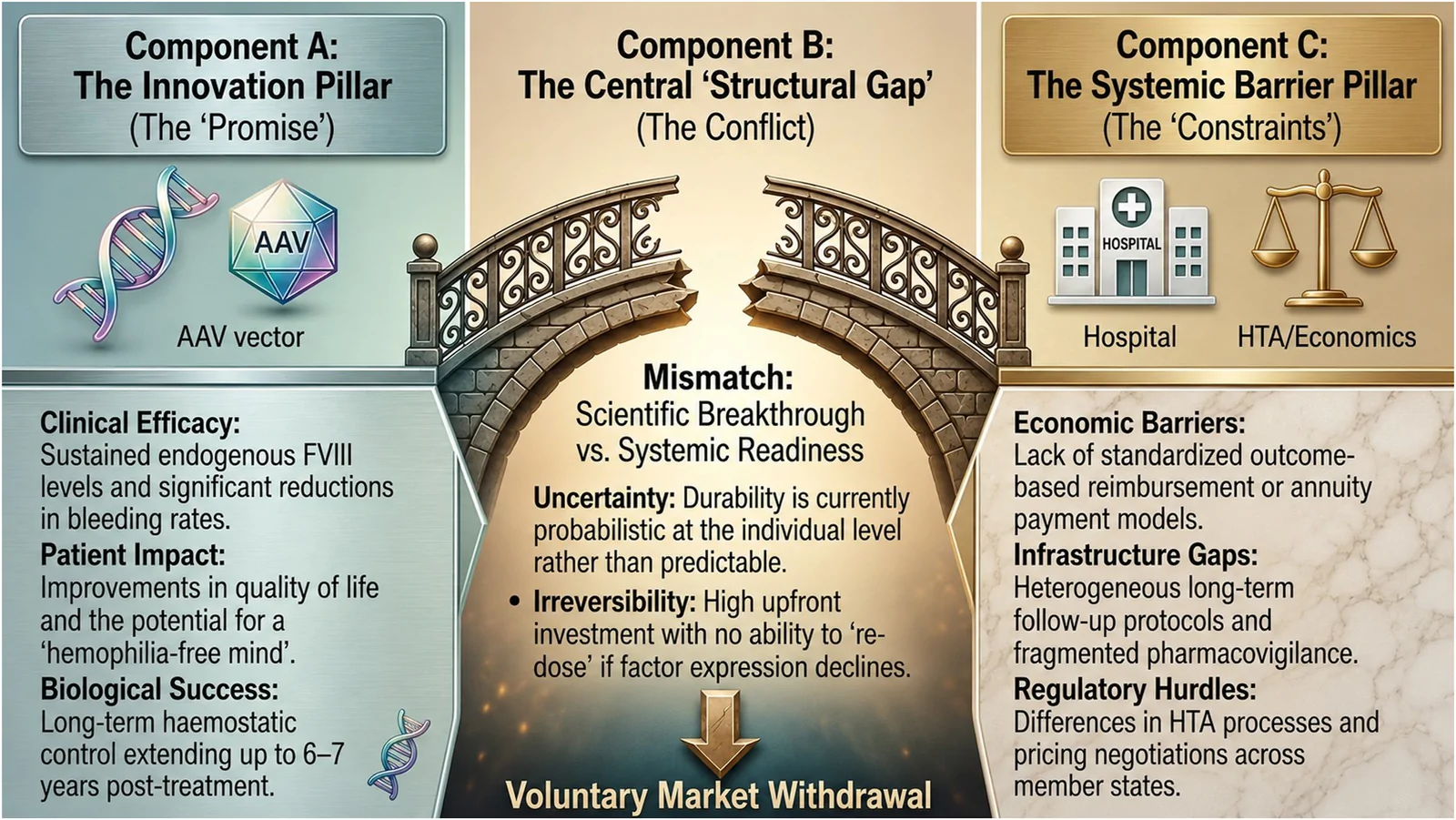
The integration gap in hemophilia A gene therapy. AAV, adeno-associated virus; FVIII, factor VIII; HTA, health technology assessment.

Despite major advances in prophylaxis, current therapies remain burdensome and do not provide a definitive cure. Patients continue to experience some breakthrough bleeding, treatment-related burden, and reduced quality of life, underscoring the unmet need that has driven the development of innovative treatment options, including gene therapy approaches [2].

Over the last few years, gene therapy has reshaped the therapeutic landscape of hemophilia A, transitioning from an experimental concept to a clinically validated intervention. Even if only a small number of patients request gene therapy, it is important to keep this option available to ensure that patients can choose, without restriction, the therapeutic approach that best adapts to their lifestyles.

In clinical trials, Roctavian has consistently demonstrated sustained increases in endogenous factor (F)VIII levels, accompanied by significant reductions in bleeding rates and improvements in quality of life [3]. Long-term follow-up studies, extending up to 6 to 7 years, confirmed durable hemostatic control in many patients, although a slight and progressive decline in FVIII expression was observed over time [4], particularly in the first year, becoming considerably less pronounced after the first year.

These biological constraints are not unexpected but highlight a key limitation of current adeno-associated viral (AAV)-based platforms: durability remains probabilistic rather than predictable at the individual level. This decline likely reflects the nonintegrating nature of AAV vectors, progressive hepatocyte turnover, and immune-mediated mechanisms affecting transgene persistence, in addition to epigenetic modifications influencing gene expression [4,5]. Despite these limitations, individual reports have demonstrated sustained reductions in bleeding episodes with a limited rate of switch back to exogenous prophylaxis, highlighting the transformative potential of gene therapy in selected patients [3]. Moreover, systematic reviews and meta-analyses further support its efficacy, reinforcing the concept of gene therapy as a paradigm shift in hemophilia care [6]. However, variability in patient response, interindividual differences in transgene expression, and the inability to readminister AAV-based therapies due to immune responses remain key limitations [7].

From a clinical standpoint, the withdrawal of Roctavian introduces a unique scenario. Unlike conventional replacement therapies, whose discontinuation results in immediate loss of therapeutic protection, gene therapy maintains its biological effect once dosed. Thus, patients already treated need to be followed up accurately. Nevertheless, the absence of commercial availability has important implications for long-term management. Gene therapy requires structured long-term follow-up at specialized, multidisciplinary centers, including monitoring of FVIII levels and management of adverse events [8].

Transient liver enzyme elevations are among the most frequently observed complications and often require corticosteroid therapy, as supported by clinical trial data and expert guidance [9], particularly in the first year. Notably, standardized long-term follow-up protocols remain heterogeneous across treatment centers [10], raising concerns about consistency in posttherapy monitoring, pharmacovigilance, and patient support.

The absence of sponsorship from a pharma company raises unresolved questions about responsibility for long-term monitoring, including funding, data collection, and pharmacovigilance infrastructure [11]. The withdrawal, therefore, shifts greater responsibility to healthcare systems to ensure continuity of care beyond commercialization. This may also increase the responsibility of regulators, who need to identify who and how these patients should be followed up and, in case of any side effects, how they should be managed.

The psychological and communicative dimensions of withdrawal are equally significant. Patients who have undergone gene therapy may experience uncertainty about the durability of the response despite reassuring long-term data. Patients who were eligible but untreated may perceive withdrawal as a lost opportunity, while those undergoing evaluation face disruption of their therapeutic pathway. This situation challenges the traditional therapeutic contract, in which innovation implies continued availability and may erode patient trust in emerging curative therapies [12]. This is a major issue, directly affecting how patients perceive future innovations, reflecting the broader tension between therapeutic promise and uncertainty [13] and emphasizing the need for clear, transparent, and empathetic communication supported by multidisciplinary care teams.

Importantly, the withdrawal of Roctavian is not an isolated event. Previous gene therapies, including alipogene tiparvovec (Glybera, uniQure biopharma B.V.) for lipoprotein lipase deficiency, Strimvelis, Orchard Therapeutics (CD34^+^ cells transduced with a retroviral vector encoding human adenosine deaminase) for adenosine deaminase-deficient severe combined immunodeficiency, and betibeglogene autotemcel (Zynteglo, bluebird bio, Inc) for transfusion-dependent β-thalassemia, have similarly faced challenges related to high costs, limited uptake, and sustainability, in some cases leading to market withdrawal or restructuring of commercialization models. Notably, in several of these cases, commercial withdrawal was followed by a marked slowdown and, in some instances, the discontinuation of further development of the same therapeutic strategy, highlighting how economic and system-level barriers may directly influence the trajectory of innovation beyond initial clinical success [14].

The Roctavian case also exposes structural challenges in the economic model of gene therapy. High upfront costs combined with uncertainty about long-term durability complicate traditional reimbursement frameworks. Cost-effectiveness analyses have yielded variable results, reflecting sensitivity to assumptions about benefit duration and the healthcare system perspective [15]. A key driver of this uncertainty is the durability of transgene expression, which remains difficult to predict and therefore complicates long-term economic evaluations and payer decision-making [15].

Unlike chronic therapies, gene therapy involves an irreversible upfront investment, amplifying payer uncertainty in the context of variable long-term durability [1,15].

Innovative reimbursement approaches, including outcome-based agreements, annuity payment models, and pay-for-performance schemes, have been proposed to address these challenges but remain inconsistently implemented across healthcare systems [15]. Furthermore, regulatory approval does not mandate continued market presence, allowing companies to withdraw products for strategic or economic reasons. Current regulatory frameworks enable approval based on limited-duration efficacy data but do not ensure sustained market availability, creating a structural gap between authorization and access [11]. Differences between regulatory agencies, such as the European Medicines Agency and Food and Drug Administration, in postmarketing requirements and pricing negotiations further contribute to variability in access and sustainability [10,11].

From a European perspective, these issues are particularly relevant. Fragmentation of health technology assessment processes, heterogeneous national reimbursement decisions, and prolonged price negotiations across European Union member states contribute to unequal access to gene therapy, amplifying disparities in patient care [1,15,16]. This raises important ethical considerations regarding equity, prioritization of healthcare resources, and the balance between one-time, potentially curative therapies and long-term chronic treatments.

At the same time, the therapeutic landscape for hemophilia A continues to evolve. Highly enhanced FVIII molecules and nonreplacement therapies (ie, bispecific antibodies and rebalancing agents) provide effective alternatives with more predictable pharmacokinetics and potentially lower economic burden [17]. Comparative analyses suggest that while gene therapy offers the possibility of long-term bleed reduction without frequent infusion, its advantages must be weighed against uncertainties in durability, cost, and patient selection [18].

Emerging innovations aim to address the current limitations of gene therapy. These advances collectively target 2 central limitations: durability and reproducibility of the response. Next-generation AAV vectors, engineered FVIII constructs with improved secretion efficiency, and alternative delivery strategies, including nonviral platforms and immune modulation approaches, may enhance durability and enable redosing in the future [19]. In parallel, the development of robust real-world evidence frameworks and international registries will be essential to better define long-term outcomes and inform clinical and policy decision-making [16].

Rather than representing a failure, the withdrawal of Roctavian should be interpreted as part of the evolution of gene therapy. It reveals the tension between innovation and implementation, emphasizing the need for robust long-term data, improved vector design, and innovative reimbursement strategies. It also underscores the importance of building healthcare infrastructure capable of supporting advanced therapies beyond their commercialization phase.

In conclusion, Roctavian embodies both the promise and the challenges of gene therapy. Its withdrawal reflects not a limitation of science but a mismatch between innovation and system readiness. Addressing this gap will require coordinated efforts among clinicians, regulators, industry, and payers.

The success of gene therapy will ultimately be determined not by its scientific feasibility, but by our capacity to integrate it into sustainable and equitable healthcare systems [1,15,16].
